# Construction and hardware validation of a transition-aware quantum kernel for pre-hypotensive PPG and ECG

**DOI:** 10.1016/j.mex.2026.104192

**Published:** 2026-10-07

**Authors:** Yevhen Darnapuk, Polina Kravchenko, Gennady Chuiko

**Affiliations:** Department of Computer Engineering, Petro Mohyla Black Sea National University, Mykolaiv, Ukraine

**Keywords:** Quantum kernel, Quantum machine learning, Photoplethysmography, Electrocardiography, Intraoperative hypotension, Quantum hardware*.*

## Abstract

Full quantum-kernel estimation requires a quadratic number of pairwise fidelity evaluations, which limits hardware studies on public cloud QPUs. We constructed paired stable and pre-hypotensive observations from VitalDB and represented each observation by its current physiological state and the change across two adjacent 60-s windows. Development-only analysis selected a 30-s prediction gap and four PPG/ECG-derived features: PPG amplitude, PPG peak width at half prominence, pulse interval, and pulse-arrival time. The Hemodynamic Transition Quantum Kernel (HTQK) encodes four state values with $R_{y}$rotations and their four corresponding changes with $R_{z}$rotations on the same four qubits, separated by an internal nearest-neighbour CX bridge. This placement avoids the cancellation that affected a fixed terminal entangler in the original compute–uncompute circuit. The locked kernel was then validated through exact, repeated finite-shot, backend-derived-noise, and physical-QPU stages. Physical validation was restricted to a deterministically selected subset of 16 development and eight holdout observations. The entangled kernel achieved holdout balanced accuracy of 0.623 (95% bootstrap CI 0.576–0.674). On *ibm_marrakesh*, 252 circuits used 148 QPU s. Relative Frobenius distortion from the exact training kernel was 0.0420 and centred kernel alignment was 0.9992. The method evaluates resource-efficient kernel preservation rather than quantum computational advantage.

## Graphical abstract

**Figure:**
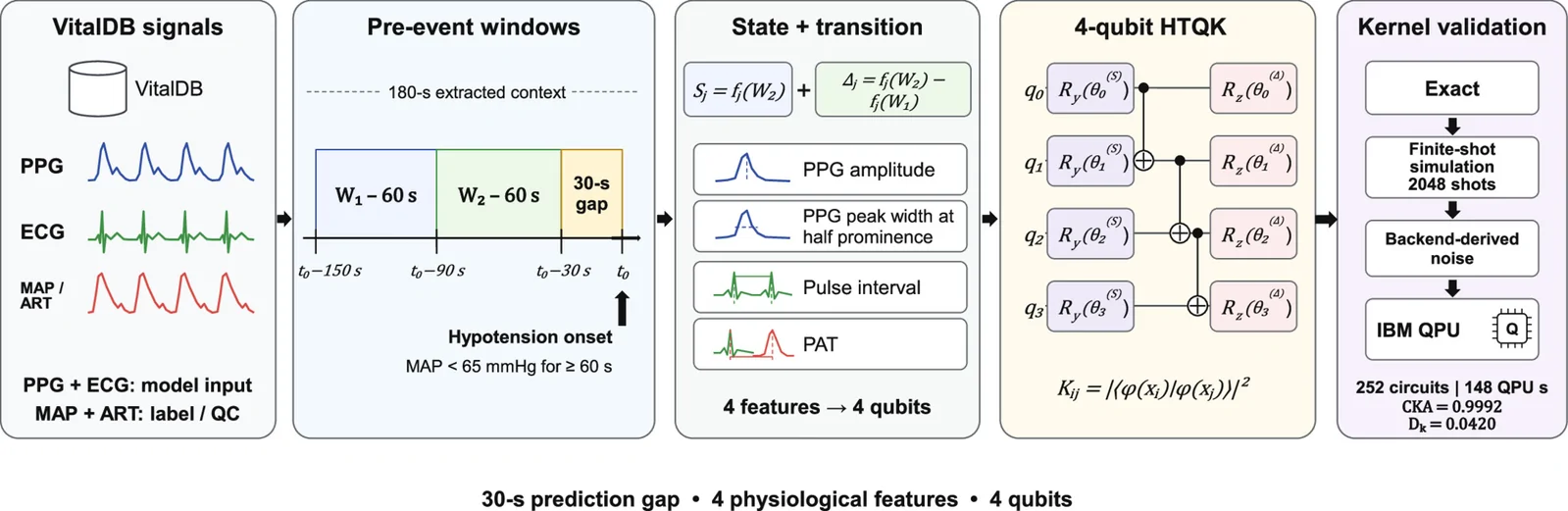

## Specifications table

**Table:** 

Subject area	Computer Science
**More specific subject area**	Quantum machine learning; biomedical signal processing
**Name of your method**	Hemodynamic Transition Quantum Kernel (HTQK) workflow
**Name and reference of original method**	Quantum feature-space and fidelity-kernel methods. Schuld M, Killoran N. Quantum machine learning in feature Hilbert spaces. Physical Review Letters. 2019;122:040,504. DOI: 10.1103/PhysRevLett.122.040504. Havlíček V, Córcoles AD, Temme K, Harrow AW, Kandala A, Chow JM, Gambetta JM. Supervised learning with quantum-enhanced feature spaces. Nature. 2019;567:209–212. DOI: 10.1038/s41586–019–0980-2.
**Resource availability**	VitalDB: DOI 10.13026/czw8-9p62. Derived cohort tables, feature matrices, kernel matrices, QPU measurements and execution metadata: 10.17632/pkschbzzk7.1.

## Background

Intraoperative arterial hypotension is a clinically important haemodynamic event, and contemporary perioperative recommendations emphasise avoiding sustained low arterial pressure in patients at risk of organ injury [1]. High-resolution non-invasive signals provide information about cardiovascular state before a pressure threshold is crossed. Photoplethysmography (PPG) contains pulse-amplitude, morphology and timing information, while an electrocardiogram (ECG) provides a cardiac timing reference from which pulse-arrival time (PAT) can be derived. PPG and ECG have consequently been used for non-invasive blood pressure estimation and in machine-learning models for intraoperative hypotension [[2], [3], [4]].

VitalDB contains synchronised intraoperative waveforms and numeric variables from 6388 surgical cases, including PPG, ECG and invasive arterial pressure [5,6]. Recent studies have used these signals in classical models for blood-pressure estimation and intraoperative hypotension prediction [[2], [3], [4]]. Quantum machine learning investigates the use of quantum computations within learning algorithms. Fidelity kernels encode classical observations as quantum states and define similarity through the squared magnitude of their overlap [7,8]. The data-reuploading classifier combines repeated input encoding with trainable circuit operations [9]. The feature map used here instead encodes physiological state and transition variables in fixed layers without trainable circuit parameters. The present experiment uses a compact four-qubit representation so that exact and noisy simulations can be compared with kernel estimates from a physical quantum processing unit (QPU) within a limited hardware budget. A four-qubit pure state is represented by 16 complex amplitudes and can be simulated directly on a classical computer. Physical execution is used here to measure agreement with the exact kernel under actual hardware conditions.

Applications to health data include electronic health records [10]. A recent systematic review found no general empirical evidence of quantum advantage in digital-health applications [11]. More recent work has applied a standard quantum kernel to ICU physiological data [12] and a customised quantum-inspired angle–distance kernel to ECG classification [13]. Other approaches address automatic quantum-kernel design [14], quantum feature selection using sparse optimisation circuits [15], and quantum SVM classification of noisy data [16]. In HTQK, physiological features are selected on development data before hardware execution, and a classical SVM uses the kernel obtained from a fixed quantum circuit. Here, the established fidelity-kernel construction is applied to paired physiological state and transition features, and the same fixed feature map is evaluated across simulation and hardware execution. Hardware validation in this study is therefore based mainly on kernel distortion, alignment, and resource use rather than classification accuracy on the deliberately small QPU subset.

HTQK customises the standard fidelity-kernel workflow by encoding four current-state values and their four within-observation changes on the same four qubits. State $R_{y}$ rotations are followed by a directed CX bridge and transition $R_{z}$ rotations. The internal placement is essential because a fixed data-independent entangler applied after all data-encoding operations cancels from the compute–uncompute overlap and cannot affect the fidelity kernel. This follows from the elementary identity $E^{†}E=I$ and is reported as an implementation correction. The validation procedure evaluates the locked feature map through exact, repeated finite-shot, backend-derived-noise, and physical-QPU stages. Deterministic subset selection and a frozen physical layout allow examination of kernel preservation without executing the complete Gram matrices on the QPU. The prediction gap, base features, scaling transformation, and SVM C were not changed after the circuit correction. The contribution is an application-specific state–transition representation and its reproducible implementation and evaluation, not a new SVM formulation or a claim of quantum advantage.

## Method details

### Method overview

VitalDB cases are screened for the required signals and for MAP-defined hypotensive events with stable controls from the same operation. Signal and event-quality checks are applied to 180-s physiological contexts. Development data are used to compare candidate pre-event gaps and four-feature combinations before the physiological representation is fixed. The selected state and transition variables are then converted to rotation angles and evaluated with exact and finite-shot fidelity kernels. A non-overlapping holdout cohort is evaluated with the fixed physiological representation, followed by hardware validation on a deterministic 24-observation subset using a frozen four-qubit QPU layout. Full-cohort exact evaluation used 534 development observations from 267 subjects and 316 holdout observations from 158 subjects. Repeated finite-shot analysis used 40 development observations from 20 subjects. Backend-noise simulation and physical-QPU evaluation used the same subset of 16 development observations from eight subjects and eight holdout observations from four subjects. Kernel distortion, alignment, and resource use are the primary method-validation endpoints; classification metrics provide a secondary application-level check Fig. 1, Fig. 2, Fig. 3.

**Fig. 1:**
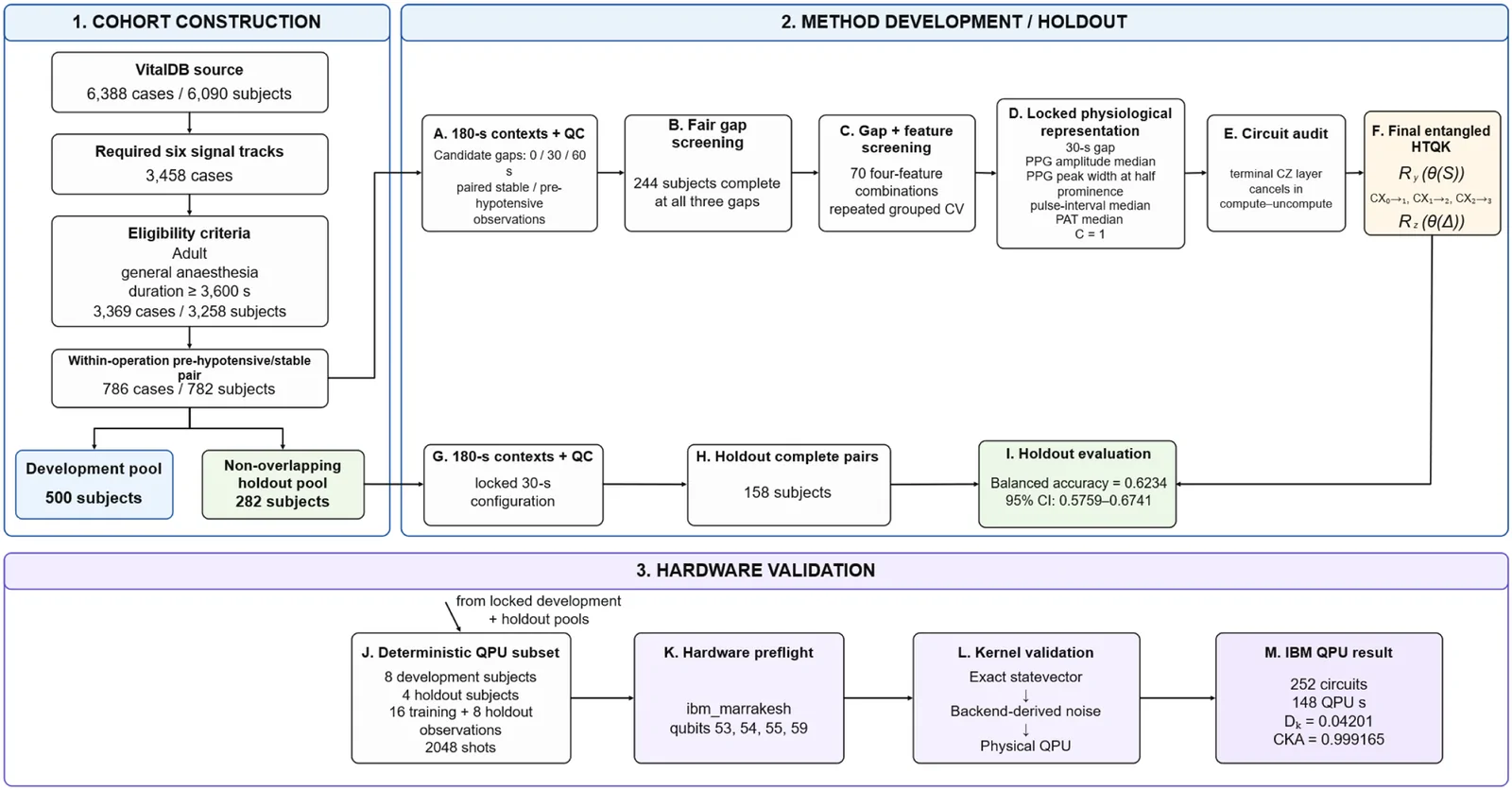
Cohort construction, development procedure, holdout evaluation, and resource-constrained hardware validation of the HTQK workflow. Gap and physiological-feature selection were restricted to the development population. After the circuit audit, the corrected entangled HTQK was evaluated on the non-overlapping holdout cohort. Physical-QPU validation used a deterministic subset of paired development and holdout observations and a frozen four-qubit IBM Marrakesh layout.

**Fig. 2:**
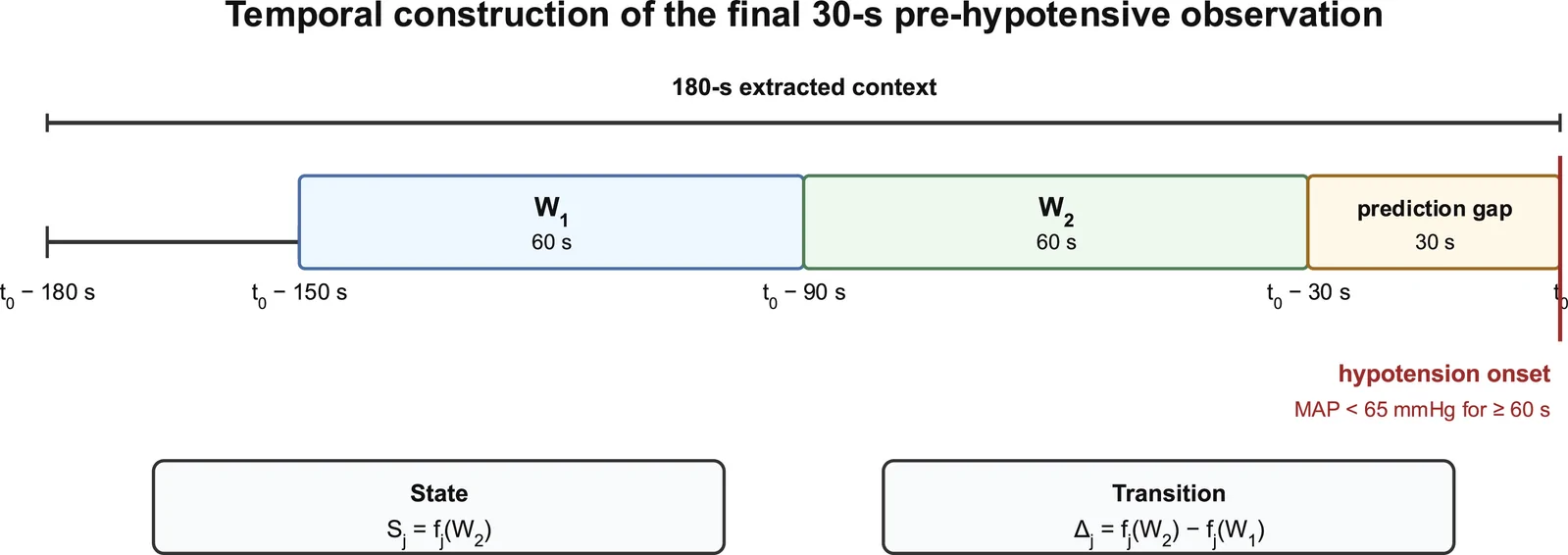
Temporal construction of a pre-hypotensive observation. Two adjacent 60-s windows form the state-transition representation. The final selected configuration leaves a 30-s gap between $W_{2}$ and the onset of the MAP-defined event.

**Fig. 3:**
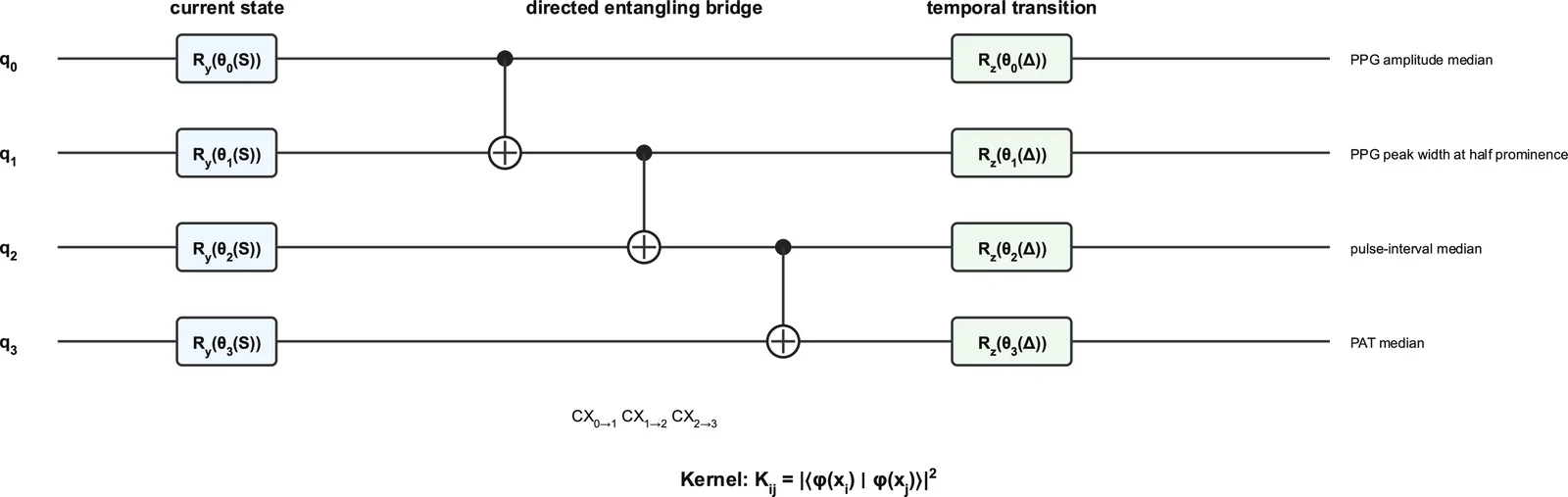
Four-qubit HTQK feature map. $R_{y}$ rotations encode the current physiological state, the directed nearest-neighbour CX chain forms the entangling bridge, and $R_{z}$ rotations encode the within-observation change.

### Dataset and initial case screening

VitalDB version 1.0.0 was used [5,6]. The database contains 6388 surgical cases corresponding to 6090 subjects. The workflow required six synchronised tracks: SNUADC/PLETH, SNUADC/ECG_II, SNUADC/ART, Solar8000/ART_MBP, Solar8000/PLETH_SPO2, and Solar8000/HR. Waveforms were loaded at 100 Hz and numeric variables at 1 Hz. The initial inventory identified 3458 cases containing all required tracks. Cases were retained if the patient was at least 18 years old, general anaesthesia was recorded, and the case duration was at least 3600 s. After these filters, 3369 cases from 3258 subjects remained.

PPG and ECG supplied the model features. Numeric MAP defined the reference event, while numeric MAP and the invasive arterial waveform were used in event-quality checks. Monitor heart rate was used for rate-consistency QC. $\text{Sp}O_{2}$ and monitor heart rate were also retained among the broader extracted descriptors, but neither entered the final four-feature HTQK representation.

### Hypotensive event and stable-control definition

A positive event was operationally defined as numeric mean arterial pressure

MAP(t)<65mmHg

for at least 60 consecutive seconds. This threshold is a research label for the present workflow and is not proposed as a new clinical definition.

Numeric MAP values below 20 mmHg or above 200 mmHg were treated as invalid measurements. VitalDB numeric tracks can contain missing positions when the source monitor reports less frequently than the requested 1-Hz grid. Interior gaps of at most 10 s were therefore interpolated, while explicitly recorded values outside the 20–200 mmHg validity interval remained invalid. A qualifying event required a complete 60-s MAP core after permitted interpolation, no explicitly invalid recorded value within that core, and MAP below 65 mmHg throughout the core. The 300-s interval immediately preceding a candidate onset had to contain at least 90% finite MAP samples after permitted interpolation and no valid MAP value below 65 mmHg. The initial event scan also required at least 90% finite MAP coverage in the 120-s interval from 180 to 60 s before onset. The earliest onset satisfying all event criteria was retained for each case.

Stable controls were selected from the same operation whenever a qualifying control was available. A candidate 120-s stable window required at least 95% finite MAP samples after permitted interpolation and MAP between 75 and 100 mmHg throughout the window. The surrounding interval extending 600 s before the window start and 600 s after the window end required at least 90% finite MAP coverage and no physiologically valid MAP value below 65 mmHg. For positive cases, a stable control also had to remain at least 600 s from the hypotensive event. Candidate starts were examined at 30-s intervals; valid windows preceding the event were preferred by temporal proximity, followed by valid post-event windows if necessary.

The scan produced 786 cases with a complete positive/stable pair, corresponding to 782 unique subjects. A further 75 cases contained a qualifying positive event but no acceptable within-operation stable control. The remaining cases consisted of 2196 external-stable/no-event cases, 311 ineligible scans and one technical retrieval error. At most one operation was retained per subject. After resolving subjects with more than one paired operation, 500 subjects were sampled for the development pool using random seed 20,260,808. The remaining 282 non-overlapping subjects formed the holdout candidate pool. No subject occurred in both groups.

### Temporal context and candidate prediction gaps

For every positive event, a 180-s context ending at the event onset $t_{0}$ was extracted:

$$\left[t_{0}-180,\;t_{0}\right)$$

An analogous 180-s stable context was constructed for the paired control. When the previously selected stable interval could not be expanded to 180 s, the closest valid 180-s stable segment in the same operation was used. We accepted a 180-s stable context only when all MAP positions were finite after permitted interpolation, no originally recorded value lay outside 20–200 mmHg, and every MAP value lay between 75 and 100 mmHg. The 600-s guard and event-separation criteria described above also applied to this expanded context.

Three candidate gaps g∈{0,30,60}s were evaluated. For a positive observation, the 120-s analysis interval ended g seconds before $t_{0}$. The interval was divided into two 60-s windows $W_{1}$ and $W_{2}$. For the final g=30s configuration,

$$W_{1}=\left[t_{0}-150,\;t_{0}-90\right)$$

$$W_{2}=\left[t_{0}-90,\;t_{0}-30\right)$$

leaving a 30-s interval between the end of the model input and hypotension onset.

For each base feature $f_{j}$, current state and temporal change were defined as

$$S_{j}=f_{j}\left(W_{2}\right)$$

$$\Delta_{j}=f_{j}\left(W_{2}\right)-f_{j}\left(W_{1}\right)$$

Thus, the transition term represents a within-observation change rather than an absolute difference between patients.

### Signal processing and quality control

PPG was band-pass filtered between 0.5 and 8 Hz. Peaks were detected with a minimum separation of 300 ms and a robust prominence derived from the median absolute deviation of the filtered signal. The local minimum preceding each pulse peak was treated as the pulse foot. Pulse amplitude and upstroke duration were calculated relative to the preceding local minimum. PPG peak width was calculated using scipy.signal.peak_widths with rel_height=0.5, corresponding to width at half prominence. For filtering only, non-finite waveform samples were filled by one-dimensional interpolation over the finite samples when at least two finite samples were present. Third-order Butterworth band-pass filters were implemented as second-order sections with zero-phase forward–backward filtering. For PPG peak detection, a robust scale $s_{\text{PPG}}$ was defined as 1.4826MAD when $\text{MAD}>10^{-9}$, and as the filtered-signal standard deviation (SD) otherwise. The prominence threshold was $p_{\text{PPG}}=\max\left\{0.35\;s_{\text{PPG}},\;10^{-6}\right\}$. The first pulse foot was sought within 1.2 s before the first peak, whereas each subsequent foot was sought between the preceding and current peaks.

ECG was filtered between 5 and 20 Hz. QRS complexes were emphasised using a derivative, squaring, and a 120-ms moving-integration procedure. Candidate complexes were refined to the strongest local ECG extremum within an 80-ms neighbourhood, and detections separated by <250 ms were merged by retaining the stronger extremum. ECG-energy candidates were required to be separated by at least 300 ms. Their prominence threshold was $p_{\text{ECG}}=\max\left\{2\left(1.4826\;\text{MAD}\right),\;0.15\;\text{SD},\;10^{-12}\right\}$, where MAD and SD were calculated from the integrated derivative energy.

PAT was calculated as the delay from each ECG R peak to the first subsequent PPG foot between 80 and 600 ms. A window required at least 20 valid PAT matches and at least 50% of the detected R peaks to be valid PPG-foot matches.

Waveforms required at least 90% finite samples. PPG- and ECG-derived heart rates had to remain between 30 and 180 beats/min. For any two compared rates $r_{a}$ and $r_{b}$, we accepted the comparison when

$$\left|r_{a}-r_{b}\right|\leq \max\left(15\;\text{beats}\;\text{mi}n^{-1},0.20\max\left(\left|r_{a}\right|,\left|r_{b}\right|\right)\right).$$

The invasive arterial waveform required a P95–P5 pulsatility of at least 10 mmHg. For positive events, the 60-s arterial waveform event core was additionally divided into 5-s segments; at least 75% of usable segments had to retain a pulsatility of at least 10 mmHg. These checks were intended to reject numerical MAP events caused by arterial-line failure or non-pulsatile artefacts.

At the final 30-s gap, 267 of the 500 development subjects retained both the stable and pre-hypotensive observations. In the non-overlapping holdout pool, 158 of 282 subjects retained both observations Table 1, Table 2, Table 3.

**Table 1:** Cohort construction.

Stage	Cases/subjects retained
VitalDB source	6388 cases / 6090 subjects
All six required signal tracks	3458 cases
Adult, general anaesthesia, duration ≥3600 s	3369 cases / 3258 subjects
Complete within-operation positive/stable pair	786 cases / 782 subjects
Development pool before 180-s QC	500 subjects
Holdout candidate pool before 180-s QC	282 subjects
Development complete pairs at selected 30-s gap	**267 subjects**
Holdout complete pairs at selected 30-s gap	**158 subjects**

**Table 2:** Development gap screening.

Gap	Best four-feature classical screen	Mean balanced accuracy	SD
0 s	PPG width, ECG HR, PAT median, PAT slope	0.6521	0.0362
**30 s**	**PPG amplitude, PPG width, pulse interval, PAT median**	**0.6345**	**0.0374**
60 s	PPG amplitude, PPG width, ECG HR, PAT median	0.6255	0.0308

**Table 3:** Locked quantum-feature transform.

Feature	State imputation	State 1st–99th percentile	Transition imputation	|Δ| 99th percentile
PPG amplitude median	32.6492	3.6753–41.1908	−0.1204	10.3621
PPG peak width at half prominence (s)	0.24374	0.14735–0.38115	−0.001716	0.066018
Pulse interval median (s)	0.880	0.540–1.270	**0**	0.3434
PAT median (s)	0.510	0.190–0.600	**0**	**0.2168**

### Physiological feature extraction

Each $W_{1}$ and $W_{2}$ segment yielded PPG amplitude median and interquartile range, PPG peak width at half prominence, PPG upstroke time, pulse-interval median, pulse-interval RMSSD and interquartile range, PPG amplitude slope, PAT median, PAT interquartile range and slope, ECG-derived heart rate, PPG-derived pulse rate, $\text{Sp}O_{2}$ median and interquartile range, and monitor heart rate. For the amplitude- and PAT-slope descriptors, we assigned the valid beat-level values evenly spaced coordinates across the 60-s window and used the median of all pairwise slopes, providing a Theil–Sen-like robust slope estimate.

Feature selection for the four-qubit method was restricted in advance to eight PPG/ECG-derived candidates with at least 95% availability: PPG amplitude median, PPG peak width at half prominence, PPG upstroke time, pulse-interval median, pulse-interval RMSSD, ECG-derived heart rate, PAT median, and PAT slope. Monitor heart rate was therefore not eligible to become a final quantum predictor.

### Development-only gap and feature screening

To compare prediction gaps fairly, gap screening used only the 244 development subjects with complete paired observations across all three candidate gaps. Every four-feature combination from the eight candidate features was evaluated:

$$\left(\begin{array}{c} 8\\ 4 \end{array}\right)=70$$

For each combination, the classical screening representation contained four state variables $S_{j}$ and their four corresponding variables $\Delta_{j}$. A class-balanced radial-basis-function support vector machine (RBF-SVM) with C=1 and γ=scale was used only for screening. Median imputation and standardisation were fitted within the training portion of every fold. Evaluation used five-fold subject-grouped cross-validation repeated five times.

The best mean balanced accuracy was 0.6521 at g=0s, followed by 0.6345 at g=30s and 0.6255 at g=60s. A gap-tie rule was specified before locking: gaps within 0.02 in balanced accuracy of the best result were treated as practically tied, and the longer gap was selected. The difference between 0 and 30 s was 0.0176, whereas the 60-s result lay 0.0266 below the best result. The final gap was therefore 30 s.

The three highest-ranked four-feature combinations at the selected 30-s gap were then evaluated using the exact quantum kernel under five-fold subject-grouped cross-validation repeated three times. C∈{0.1,1,10,100} was evaluated for the precomputed-kernel SVM. The combination containing PPG amplitude median, PPG peak width at half prominence, pulse-interval median and PAT median produced the highest initial quantum-kernel result, with C=1. The gap, four base features and C were then locked. With the selected feature combination, the state-only and transition-only quantum kernels were each evaluated over C∈{0.1,1,10,100} using five-fold subject-grouped cross-validation repeated three times. Selection by mean development balanced accuracy was performed independently for each kernel, yielding C=10 for both ablations. The combined kernel retained its locked C=1, including after the circuit correction.

**Scaling to quantum rotation angles**

Scaling parameters were fitted only on the development cohort. Missing state and transition values were median-imputed using development values.

For state variable $S_{j}$, the development 1st and 99th percentiles $l_{j}$ and $h_{j}$ defined

$$\theta_{j}^{\left(S\right)}=\pi \;\text{clip}\left(\frac{S_{j}-l_{j}}{h_{j}-l_{j}},0,1\right)$$

For the transition variable $\Delta_{j}$, the 99th percentile $a_{j}$ of $|\Delta_{j}|$ defined

$$\theta_{j}^{\left(\Delta \right)}=\pi \;\text{clip}\left(\frac{\Delta_{j}}{a_{j}},-1,1\right)$$

clip(x,a,b)=min(max(x,a),b)

The fitted values were subsequently applied unchanged to holdout and QPU-subset samples.

**Quantum feature map and fidelity kernel**

The feature map reuses each qubit for a state angle and the corresponding transition angle. It differs from a static angle-encoding map in the ordered state–bridge–transition construction, not in the individual rotation or CX gates. The final HTQK circuit uses four qubits. The current state is encoded first:

$$U_{S}\left(S\right)=\prod_{j=0}^{3}R_{y}^{\left(j\right)}\left(\theta_{j}^{\left(S\right)}\right)$$

A directed nearest-neighbour entangling bridge is then applied:

$$E_{\text{CX}}=CX_{2\to 3}\;CX_{1\to 2}\;CX_{0\to 1}$$

The rightmost operator acts first, corresponding to the circuit order $CX_{0\to 1}$, $CX_{1\to 2}$, and $CX_{2\to 3}$.

Transition information is encoded after the bridge:

$$U_{\Delta }\left(\Delta \right)=\prod_{j=0}^{3}R_{z}^{\left(j\right)}\left(\theta_{j}^{\left(\Delta \right)}\right)$$

$$U_{\text{HTQK}}\left(S,\Delta \right)=U_{\Delta }\left(\Delta \right)\;E_{\text{CX}}\;U_{S}\left(S\right)$$

The $R_{y}$ rotations encode state values in the computational-basis amplitudes, while the subsequent $R_{z}$ rotations encode transition values as relative phases in the prepared superposition. A single directed nearest-neighbour CX chain connects the four qubits with three CX gates, keeping the entangling operation shallow. These choices specify a compact representation; their optimality was not established. A fixed circuit was used to examine a specified physiological representation consistently across validation stages. Automatic circuit optimisation, such as the approach of Incudini and colleagues [14], would introduce an additional development-stage architecture search, which was outside the scope of this study.

For observation x, the resulting state is |ϕ(x)〉. It is defined by

$$\left|\phi \left(x_{i}\right)\rangle =U_{\text{HTQK}}\left(S_{i},\Delta_{i}\right)\right|0\rangle^{⊗4}.$$

Kernel elements were evaluated through compute-uncompute circuits. The feature map for the second observation is followed by the inverse feature map for the first observation; the probability of measuring |0000〉 is the fidelity estimate.

$$K_{ij}\&={\left|\langle \phi \left(x_{i}\right)|\phi \left(x_{j}\right)\rangle \right|}^{2}$$

$$={\left|{\langle 0{|}^{⊗4}U_{\text{HTQK}}^{†}\left(S_{i},\Delta_{i}\right)U_{\text{HTQK}}\left(S_{j},\Delta_{j}\right)|0\rangle }^{⊗4}\right|}^{2}$$

$$=P\left(0000|x_{i},x_{j}\right).$$

### Circuit audit and entanglement correction

The first implementation placed a data-independent CZ chain after both state and transition rotations. Hardware preflight revealed that representative compute-uncompute circuits transpiled to zero two-qubit operations. Algebraically, if the feature map is written as

$$U_{\text{old}}\left(x\right)=E\;L\left(x\right)$$

where E is the same terminal entangling unitary for every input, then

$$U_{\text{old}}^{†}\left(x\right)U_{\text{old}}\left(y\right)=L^{†}\left(x\right)E^{†}EL\left(y\right)=L^{†}\left(x\right)L\left(y\right)$$

The terminal entangler therefore has no effect on the fidelity kernel. Removing the terminal CZ layer gives a separable reference map with the same fidelity kernel as the initial implementation. More generally, a data-independent unitary placed as the final operation in every feature map is unidentifiable from a compute–uncompute fidelity kernel and must not be counted as an effective entangling layer. This finding was treated as a structural circuit defect rather than a performance-tuning opportunity. The terminal layer was replaced once by the CX bridge between the state and transition rotations described above. The 30-s gap, four selected features, scaling transformation and C=1 were not modified.

The separable reference map yielded mean development balanced accuracy 0.6436 (SD 0.0290), whereas the corrected entangled map yielded 0.6198 (SD 0.0286). No further tuning was performed. The pre-correction method lock and separable holdout outputs were preserved separately in the released circuit-audit materials. The corrected circuit was then evaluated once on the existing non-overlapping holdout cohort.

### Classical baselines

Classical models used the same four locked base features. Separate representations contained state variables only, transition variables only, or the concatenated state+transition vector. Logistic regression, linear SVM and RBF-SVM candidates were evaluated using five-fold subject-grouped cross-validation repeated five times. For logistic regression and the linear SVM, C∈{0.1,1,10,100} was evaluated. For the RBF-SVM, the same C grid and γ∈{scale,0.1,1} were evaluated. The best model for all three representations was the class-balanced RBF-SVM with C=1 and γ=scale. Across the 25 grouped folds, mean balanced accuracy was 0.5966 (SD 0.0304) for state, 0.5922 (SD 0.0398) for transition, and 0.6353 (SD 0.0253) for the combined state-plus-transition representation.

### Exact and holdout evaluation

The holdout cohort contained 158 subjects, each contributing one stable and one pre-hypotensive observation. It was not used to select the prediction gap, physiological features, feature scaling, or SVM C. Balanced accuracy, sensitivity, specificity, F1 score, Matthews correlation coefficient (MCC), area under the receiver operating characteristic curve (AUROC), and average precision (AP) were calculated. AP was computed using sklearn.metrics.average_precision_score. For balanced accuracy, 95% confidence intervals were obtained from 1000 subject-level bootstrap resamples. Subjects were sampled with replacement, with each subject’s stable and pre-hypotensive observations retained as a pair.

The initial separable quantum map had already been evaluated on this cohort before the cancellation of the terminal entangling layer in the compute–uncompute circuit was identified. After the structural correction, the entangled circuit was evaluated once without changing the prediction gap, feature set, scaling transformation, or SVM C. Accordingly, the 158-subject set is described throughout as a non-overlapping holdout cohort rather than as an untouched confirmatory cohort.

### Finite-shot analysis and shot selection

Before physical execution, finite-shot sampling was evaluated on a deterministic 20-subject development microcohort. Within the PRE observations, we calculated Euclidean distances in the locked eight-dimensional angle space. We selected the observation closest to the centroid first and then iteratively selected the observation with the largest distance to its nearest already selected observation. Ties followed the existing row order. Both paired observations were retained, producing 40 samples. The exact statevector kernel was compared with 30 independently sampled kernels at 256, 512, 1024 and 2048 shots.

Two kernel-level measures were used. Relative Frobenius distortion was

$$D_{K}\left(K,K_{\text{exact}}\right)=\frac{∥K-K_{\text{exact}}{∥}_{F}}{∥K_{\text{exact}}{∥}_{F}}$$

Centered kernel alignment (CKA) was calculated after double-centering the two Gram matrices:

$$\text{CKA}\left(K_{1},K_{2}\right)=\frac{{\langle HK_{1}H,\;HK_{2}H\rangle }_{F}}{∥HK_{1}H{∥}_{F}∥HK_{2}H{∥}_{F}}$$

where

$$H=I-\frac{1}{n}11^{T}.$$

The smallest shot count within 0.01 CKA of the maximum and within 0.01 distortion of the minimum was to be selected. The corrected entangled circuit required 2048 shots under this rule.

### QPU microcohort construction

Full QPU estimation of the development and holdout Gram matrices was avoided. Development and holdout subjects were selected independently by the same centroid-then-farthest-point procedure used for finite-shot subset selection, applied to PRE observations in the locked eight-dimensional angle space. Eight development subjects and four holdout subjects were selected, and the paired stable observation for each subject was retained. This produced 16 development and eight holdout observations. The 16-observation training matrix required $\frac{16(16-1)}{2}=120$ off-diagonal fidelity circuits. The eight holdout observations required 8×16=128 cross-kernel circuits. Four self-fidelity sentinels brought the physical workload to 120+128+4=252 circuits.

The deterministic subset was intended to test preservation of the locked kernel on the selected observations; it was not used to approximate or retrain the full-cohort classifier. The selection procedure does not guarantee population representativeness or formal feature-space coverage. Sensitivity to subject composition was examined by removing each subject’s stable and pre-hypotensive observations together from the existing exact and measured kernels. Eight training-subject deletions and four holdout-subject deletions were evaluated separately. Training-kernel distortion and CKA were recalculated after each training-subject deletion, and cross-kernel distortion and Pearson correlation were recalculated after both types of deletion. CKA was recalculated after centring each retained training matrix. No additional hardware measurements were acquired. Classification on its eight holdout observations is reported only as a decision-preservation check, namely, whether hardware-induced kernel changes alter the predictions of the corresponding exact subset model.

### Backend and physical-qubit preflight

Operational IBM QPUs accessible during preflight were screened for calibrated connected four-qubit paths. Candidate paths were first ranked by current calibration quality, and the ten highest-ranked paths on each backend were retained for transpilation. Three representative compute-uncompute circuits were transpiled on each shortlisted path using optimisation level 3, physical-qubit placement fixed to the candidate path and transpiler seed 20,260,808. Candidates whose representative circuits contained no two-qubit operations after transpilation were rejected.

Among the surviving paths, the path with the largest mean calibration-based transpiled-circuit success proxy was selected. Ties were resolved, in order, by smaller maximum readout error, smaller mean two-qubit error, smaller maximum edge error, smaller representative depth, and then smaller operation count. Queue length was recorded but was not used for selection.

The final backend was ibm_marrakesh, with physical qubits 53, 54, 55 and 59. At preflight, their mean readout error was 0.003906 and maximum readout error was 0.005005. The three corresponding CZ calibration errors were 0.001292, 0.001753 and 0.001883. Representative compute-uncompute circuits had depths of 26–28 and retained six two-qubit operations after optimisation-level-3 transpilation, confirming that the entangling bridge survived compilation. The transpiler seed was 20,260,808. The backend and physical layout were frozen after preflight and reused for both backend-derived noise simulation and QPU execution.

### Backend-noise simulation

A Qiskit Aer simulator was created from the frozen IBM backend and executed using the same physical layout, 2048 shots and transpiled circuits planned for hardware. Raw and positive-semidefinite-projected kernel matrices were stored separately. PSD projection was included as a contingency for noisy empirical Gram matrices; the actual backend-noise kernel had no negative eigenvalues.

### Physical IBM QPU execution

Hardware evaluation used Qiskit Runtime SamplerV2 in job mode. No error mitigation was applied. The 252 circuits were executed at 2048 shots on the frozen ibm_marrakesh layout. A 16-circuit pilot was submitted first and consumed 11 QPU s. The remaining circuits were submitted in four additional jobs. The five jobs used 11, 37, 37, 37 and 26 QPU s, respectively, for a total of 148 QPU s. Every required circuit returned exactly 2048 samples, with no duplicated kernel entries. The training-kernel diagonal was fixed to its theoretical value of one. The four separately executed self-fidelity sentinels were retained as an independent measurement sanity check and were not substituted into the Gram-matrix diagonal. Consequently, the reported training-kernel distortion contains no diagonal error, while its reference norm includes the unit diagonal. CKA also depends on this reconstruction convention through matrix centring; fixing the diagonal does not have a universally predictable effect on CKA. The four sentinels do not provide experimental estimates of all 16 training diagonals. Additional comparisons evaluated training-kernel distortion using only measured off-diagonal entries, with both the error norm and reference norm restricted to those entries. For the four-observation principal submatrix with measured self-fidelities, distortion and CKA were compared using either unit or measured diagonals, while keeping the off-diagonal entries unchanged.

### Software and reproducibility

The final framework used Python with VitalDB 1.7.2, NumPy 2.x, pandas 2.x, SciPy 1.x and scikit-learn 1.x. The quantum components used Qiskit 2.5.1, Qiskit Machine Learning 0.9.0, Qiskit IBM Runtime 0.48.0 and Qiskit Aer 0.17.2 [17]. The main random seed was 20,260,808. Cohort construction, repeated cross-validation, finite-shot repetitions, physical-subset selection and transpilation used deterministic seeds recorded by the framework. Original VitalDB waveform files are not redistributed in the derived-data package. The released Mendeley Data record contains cohort and QC tables, derived physiological features, method locks, development and holdout validation outputs, exact and empirical kernels, backend-noise measurements, raw QPU counts and execution metadata (DOI: 10.17632/pkschbzzk7.1).

## Method validation

The method was validated at three levels. Numerical validation measured finite-shot repeatability against the exact statevector kernel. Hardware validation examined preservation of the locked kernel under backend-derived noise and physical-QPU execution using relative Frobenius distortion, centred kernel alignment, eigenvalue checks, cross-kernel correlation, and self-fidelity sentinels. Application-level validation examined whether the locked kernel supported discrimination between stable and pre-hypotensive observations on the non-overlapping holdout cohort. Kernel preservation and resource use were the primary method-validation outcomes; classification metrics were secondary and were not interpreted as evidence of quantum advantage.

### Application-level validation on the locked holdout cohort

The corrected entangled HTQK yielded mean development balanced accuracy 0.6198 (SD 0.0286) across 15 subject-grouped folds. On the 158-subject holdout cohort, balanced accuracy was 0.6234, with a subject-level bootstrap 95% CI of 0.5759–0.6741. On holdout data, the classical state+transition RBF-SVM and the entangled HTQK both achieved balanced accuracy 0.6234. AUROC was 0.6593 and 0.6530, respectively. These results provide no evidence that the quantum kernel improves predictive performance over the classical RBF-SVM.

The classical RBF-SVM models used C=1and γ=scale. Development cross-validation selected C=10for the state-only and transition-only quantum kernels, whereas the separable reference and final entangled HTQK kernels retained C=1. Table 4 provides an application-level check of the locked binary-discrimination pipeline; numerical and hardware kernel preservation remain the primary validation endpoints.

**Table 4:** Application-level holdout validation of the locked classical and quantum representations.

Model	Balanced accuracy	Sensitivity	Specificity	F1	MCC	AUROC	AP
Classical state RBF-SVM	0.6076	0.5696	0.6456	0.5921	0.2158	0.6391	0.6307
Classical transition RBF-SVM	0.5633	0.4051	0.7215	0.4812	0.1334	0.5787	0.5461
Classical state+transition RBF-SVM	0.6234	0.5633	0.6835	0.5993	0.2486	0.6593	0.6593
Separable reference quantum kernel	0.6108	0.5633	0.6582	0.5914	0.2225	0.6556	0.6710
Final entangled HTQK	0.6234	0.5823	0.6646	0.6073	0.2477	0.6530	0.6572
Quantum state kernel	0.5981	0.4810	0.7152	0.5448	0.2018	0.6305	0.6149
Quantum transition kernel	0.5696	0.4114	0.7278	0.4887	0.1468	0.6029	0.5921

Fig. 4 compares the development means with the holdout estimates and their uncertainty.

**Fig. 4:**
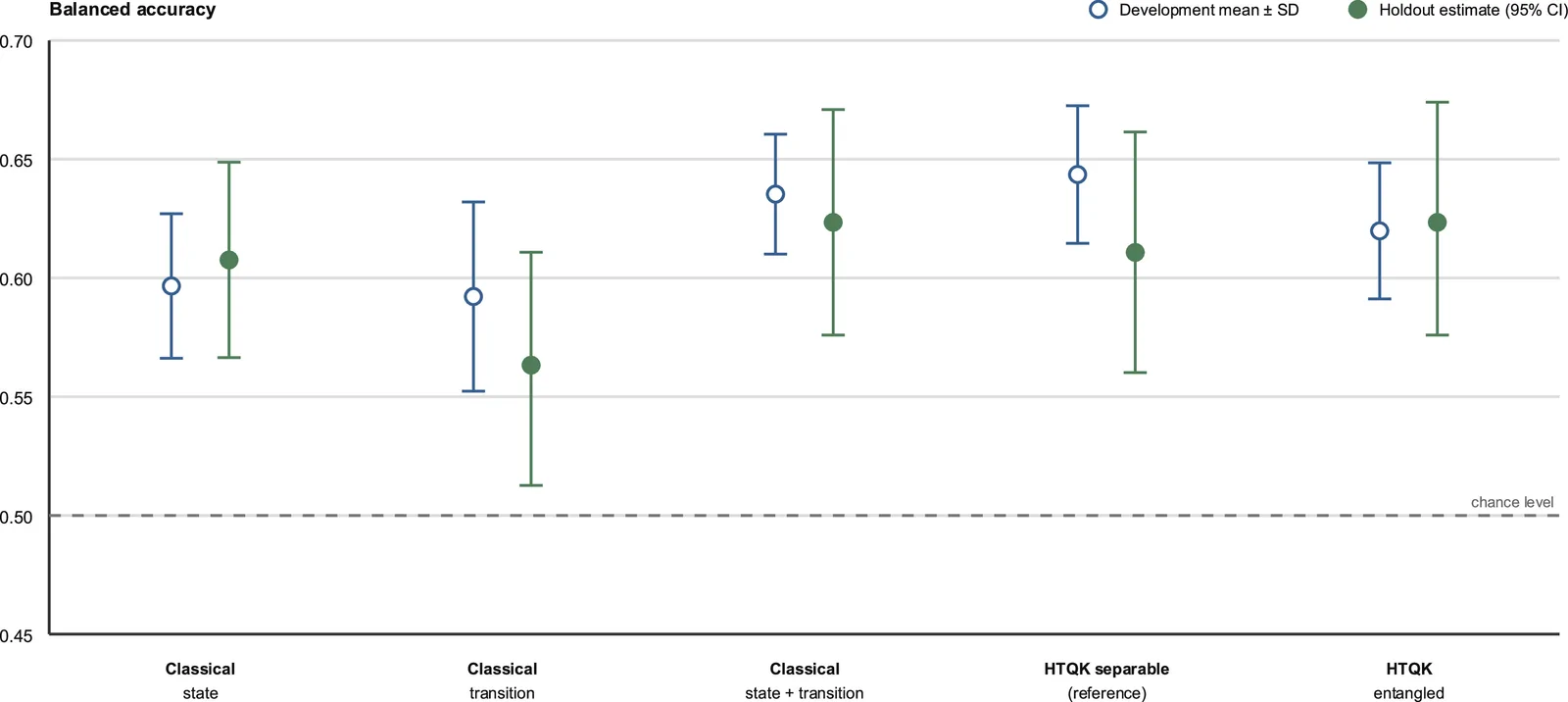
Development and holdout balanced accuracy for the locked classical and quantum representations. Development error bars show ±1 SD across 25 grouped folds for the classical models and 15 grouped folds for the quantum kernels. Holdout error bars show 95% subject-level bootstrap confidence intervals from 1000 resamples. The separable reference kernel is equivalent to the initial kernel, and the entangled kernel is the corrected HTQK.

### Numerical validation under finite-shot sampling

Numerical repeatability was assessed by comparing 30 independently sampled kernels at each shot count with the exact statevector kernel. Table 5 shows the monotonic improvement in kernel preservation as the shot count increased.

**Table 5:** Repeated finite-shot preservation of the corrected entangled kernel (30 repetitions per shot count).

Shots	Mean CKA	SD CKA	Mean $D_{K}$	SD $D_{K}$
256	0.996163	0.000193	0.069214	0.001710
512	0.998055	0.000081	0.049194	0.000975
1024	0.999028	0.000061	0.034705	0.001085
**2048**	**0.999518**	**0.000024**	**0.024454**	**0.000571**

The 2048-shot setting satisfied the predefined plateau criterion and was therefore used for the backend-noise and physical-QPU stages.

### Validation under backend-derived noise

Against the exact training kernel, the backend-derived kernel had $D_{K}=0.02305$, CKA=0.999711, and a minimum eigenvalue of 0.1662. No eigenvalue was below the numerical tolerance of -10^-10^, so PSD projection did not change the kernel.

### Physical-QPU kernel validation

Against the exact training kernel, physical execution yielded $D_{K}=0.04201$, CKA=0.999165, and a minimum eigenvalue of 0.2093. Distortion was 0.03427 in the stable training subkernel and 0.01588 in the pre-hypotensive training subkernel. The 128 exact and hardware holdout-to-training kernel entries had Pearson correlation r=0.99868. The four self-fidelity sentinels had a mean fidelity of 0.98193 and a minimum fidelity of 0.97754.

Across the eight training-subject deletions, training-kernel distortion ranged from 0.03057 to 0.04339 and CKA from 0.999093 to 0.999471, with unit diagonals retained. Cross-kernel distortion ranged from 0.05050 to 0.05703, compared with 0.05319 for the original subset, and Pearson correlation ranged from 0.99824 to 0.99898. Across the four holdout-subject deletions, cross-kernel distortion ranged from 0.05202 to 0.05524 and correlation from 0.99829 to 0.99904. High alignment and correlation were retained across these deletions, although distortion varied with subject composition. Restricting the training-kernel comparison to measured off-diagonal entries gave a relative distortion of 0.06898, compared with 0.04201 for the matrix with unit diagonals. In the four-observation submatrix with measured self-fidelities, replacing unit diagonals with measured diagonals changed distortion from 0.04595 to 0.04872 and CKA from 0.998901 to 0.999274. These submatrix results do not establish the effect of measured diagonals on the complete 16-observation training matrix Table 6.

**Table 6:** Kernel-preservation comparison on the QPU subset.

Stage	Shots	Relative training-kernel distortion	CKA	Raw negative eigenvalues
Exact statevector	—	0	1	0
Backend-derived noise	2048	0.02305	0.999711	0
**IBM Marrakesh QPU**	**2048**	**0.04201**	**0.999165**	**0**

Training-kernel metrics use diagonals fixed to one; self-fidelity measurements are reported separately. The finite-shot and backend/QPU tables were generated on different predefined microcohorts, so their distortion values do not form a paired decomposition of sampling and hardware error. The backend-derived-noise and QPU values, by contrast, refer to the same frozen hardware subset and can be compared directly. On that subset, the locked kernel geometry remained closely aligned from exact statevector evaluation through backend-derived noise to physical execution. Fig. 5 visualises the three training kernels and the exact-to-QPU cross-kernel comparison.

**Fig. 5:**
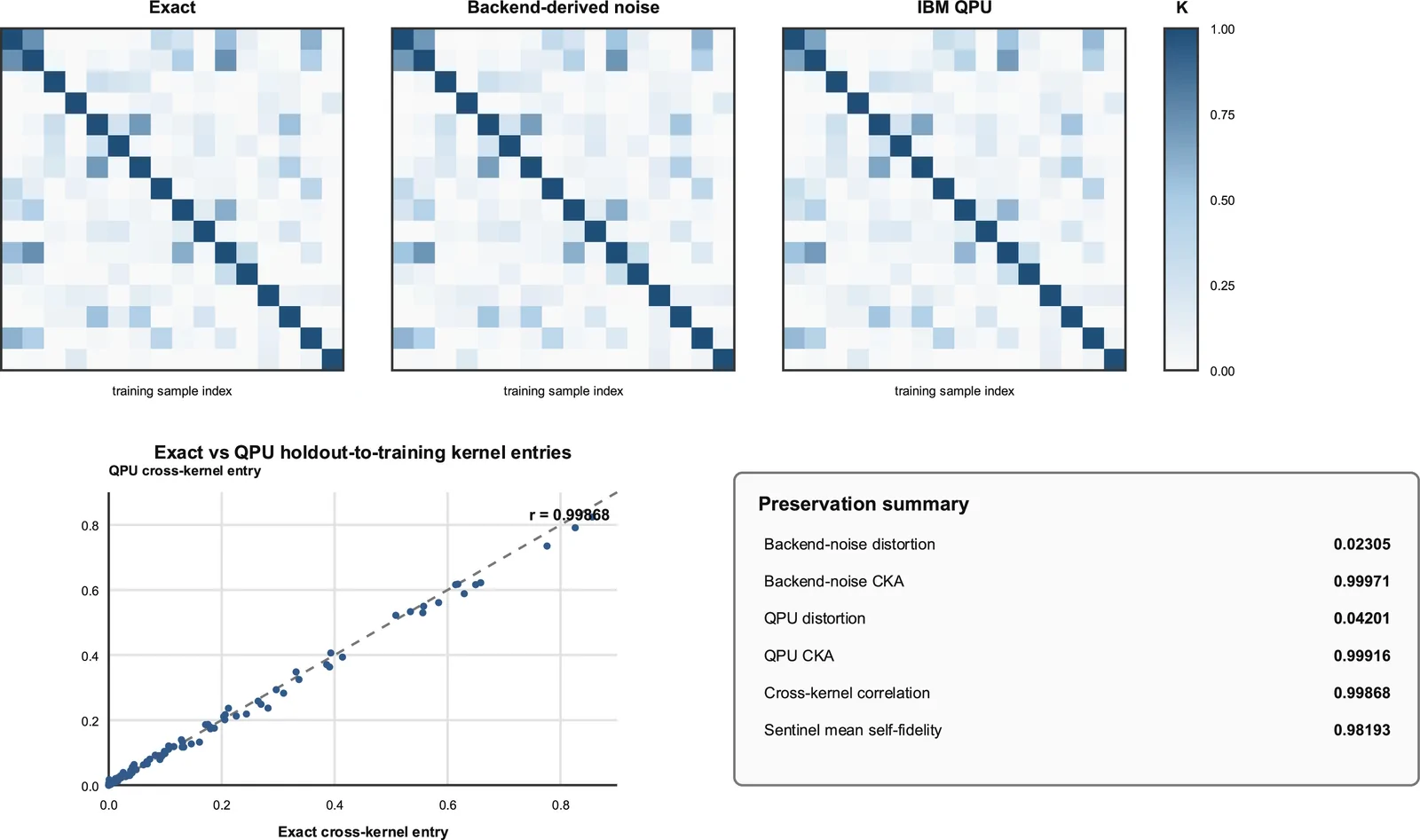
Kernel preservation for the deterministic QPU subset. The top panels show the exact, backend-noise, and IBM QPU training kernels in the same fixed sample order; the lower-left panel compares the 128 exact and QPU holdout-to-training kernel entries. Training-kernel diagonals were fixed to one, and the four independently measured self-fidelity sentinels were analyzed separately. The exact-to-QPU cross-kernel correlation was r=0.99868.

### Descriptive QPU-subset classification

The physical hardware subset contained only eight holdout samples, so its classification statistics are descriptive rather than estimates of clinical predictive performance. The exact and hardware subset kernels both produced balanced accuracy 0.625, sensitivity 0.75, specificity 0.50, F1 0.667 and MCC 0.258, and all eight predictions agreed. The comparison checks only whether the observed hardware perturbation changed the decisions of the exact subset model; in this experiment, it did not.

### Validation workload and resource efficiency

The full 534-observation development Gram matrix would require 142,311 off-diagonal fidelity circuits, while the 316×534 holdout cross-kernel would require 168,744 circuits. Complete full-matrix hardware validation would therefore require 311,055 kernel circuits. More generally, direct pairwise estimation for N development observations and M holdout observations requires $\frac{N(N-1)}{2}+MN$ kernel circuits when training diagonals are fixed to one. At s shots per circuit, this requires $s[\frac{N(N-1)}{2}+MN]$ circuit executions, excluding sentinels and additional repetitions. The hardware subset evaluation instead used 120 training-kernel circuits, 128 holdout-to-training circuits, and four self-fidelity sentinels, giving 252 circuits at 2048 shots. The 99.919% reduction in circuit count results from restricting physical validation to the predefined subset; it does not reduce the cost of estimating the complete kernel matrices, which were evaluated by exact simulation. The five jobs used 148 s of recorded QPU time. This reduction applies to kernel-preservation validation only; the deterministic subset is not proposed as a replacement for full-cohort model training.

## Limitations

The method was developed and evaluated using a single public perioperative dataset collected at one institution. VitalDB itself notes that its population is from a single institution and predominantly one racial group [5], so physiological generalisation to other centres and populations is not established. Differences in patient populations, surgical practice, monitoring equipment and signal acquisition may change the distributions of the extracted features. Transfer of the selected four-feature representation and development-fitted transformations to another dataset or institution remains untested.

The MAP<65mmHg for 60 s event rule is an operational research definition. MAP and the invasive arterial waveform were used as reference and QC signals, but the method has not been prospectively evaluated as a clinical warning system. Stable controls were drawn from the same operation to reduce between-patient variability, although within-operation interventions, anaesthetic depth, vasoactive treatment, fluid administration and time-dependent physiological changes may still confound the comparison.

Feature engineering is deliberately compact. Four hand-engineered PPG/ECG-derived variables cannot represent the full information available in raw waveforms. The development procedure also selected the temporal gap and feature combination on the same development population, although subject-grouped repeated cross-validation and a separate holdout population were used to limit optimistic evaluation.

The circuit audit uncovered an implementation issue after the initial quantum kernel had already been evaluated on the holdout cohort. The correction was triggered by algebra and transpilation, not by adverse predictive performance: the gap, features, scaling and C remained unchanged, and the corrected entangled circuit was evaluated once. Nevertheless, this sequence means that the corrected-circuit holdout result should not be described as completely untouched post-audit validation.

The final four-qubit circuit is readily simulable on a classical computer. No computational speed-up, quantum advantage or clinical utility is claimed. Increasing the number of qubits would not itself establish computational advantage. The effects of larger feature maps, increased circuit depth and the shot counts needed to maintain kernel accuracy were not evaluated. The QPU subset was intentionally small, with 16 training and eight holdout observations, and classification statistics from that subset are descriptive only. The preservation results are conditional on these selected observations and this hardware execution; they do not establish robustness across alternative subsets, larger cohorts or repeated hardware runs. The subject-deletion ranges describe sensitivity within the measured subset and are not confidence intervals. Because deletion changes both matrix size and subject composition, this analysis does not estimate variability across independently selected subsets of the original size. Hardware calibration is also time-dependent; the reported physical-qubit errors describe the preflight snapshot for this execution rather than permanent properties of the processor. No QPU error mitigation was applied, and the backend-derived noise model is only an approximation of physical hardware behaviour.

## Ethics statements

This study is a secondary computational analysis of the publicly available, anonymised VitalDB dataset. The original VitalDB data acquisition and public disclosure were approved by the Institutional Review Board of Seoul National University Hospital (H-1408-101-605), and written informed consent was waived because of data anonymity [5,6].

## CRediT author statement

Y.D., P.K., and G.C. contributed equally to the conceptualisation, methodology, simulation design, data generation, analysis, and interpretation of the results. All three authors contributed to drafting, reviewing, and editing the manuscript and approved the final version.

## Declaration of competing interest

The authors declare that they have no known competing financial interests or personal relationships that could have appeared to influence the work reported in this paper.

## Data Availability

Data associated with this article have been deposited in a publicly available repository.
